# Describing terminologies and discussing records: More discoveries of facultative vivipary in the genus *Hedychium* J.Koenig (Zingiberaceae) from Northeast India

**DOI:** 10.3897/phytokeys.96.23461

**Published:** 2018-03-15

**Authors:** Ajith Ashokan, Vinita Gowda

**Affiliations:** 1 Tropical Ecology and Evolution (TrEE) Lab, Department of Biological Sciences, Indian Institute of Science Education and Research- Bhopal, Madhya Pradesh, India- 462066

**Keywords:** Facultative vivipary, gingers, Meghalaya, Nagaland, phenology, pseudovivipary, recalcitrant seeds

## Abstract

The authors introduce the term facultative vivipary for the first time in gingers and elaborate on this reproductive strategy. Four new observations of facultative vivipary are reported in the genus *Hedychium* which were discovered during botanical explorations by the authors in Northeast India (NE India) over the past three years. The viviparous taxa are *H. marginatum* C.B.Clarke, H. speciosum var. gardnerianum (Ker Gawl.) Sanoj & M.Sabu (previously, *H. gardnerianum* Sheppard ex Ker Gawl.), *H. thyrsiforme* Buch.-Ham. ex Sm. and *H. urophyllum* G.Lodd. The authors also attempt to summarise the occurrence of vivipary in the family Zingiberaceae from published reports and to clarify a taxonomic misidentification in a previously known report of vivipary in *Hedychium elatum*.

## Introduction

Vivipary in plants is a heterogeneous term that describes a unique and rare reproductive strategy where seedlings are precociously produced while still on the maternal parent (Goebel 1905). With the discovery of several examples of germination on the parent plant in various angiosperm families, vivipary has become a biologically complex term because it now accommodates both sexual (true vivipary and cryptovivipary, see Elmqvist and Cox 1996) as well as asexual reproductive (pseudovivipary) strategies in plants (Farnsworth 2000).

True vivipary refers to vivipary *sensu stricto* and it is defined as penetration of a sexually reproduced embryo through the fruit pericarp and the resultant dispersal of this seedling (Cota-Sánchez 2004). Contrastingly, cryptovivipary is defined as vivipary wherein the seedling does not penetrate the fruit pericarp (Cota-Sánchez and Abreu 2007), while pseudovivipary is defined as production of apomictic or asexual propagules (like bulbils) on the parent plant (Law et al. 1983, Ofir and Kigel 2014). Thus sexually reproduced progeny (seed) is an integral part of the definition of vivipary and should not be confused with pseudovivipary which refers to production of apomictic or asexual plantlets or bulbils (Poulsen and Nordal 2005).

Within the family Zingiberaceae, vivipary is rarely discussed as an important reproductive strategy, except in *Hedychium elatum* R.Br. by Bhadra et al. (2013) (taxonomic misidentification discussed later). However, vivipary has been recorded in taxonomic descriptions such as in *Camptandra latifolia* Ridl. (Ridley 1899), in natural history observations (*Hedychium gardnerianum* Sheppard ex Ker Gawl.; Djeddour et al. 2012) and in pollination studies (*Alpinia mutica* Roxb.; Aswani and Sabu 2015). In contrast, pseudovivipary is very common in the form of bulbils and it has been recorded in at least six genera within Zingiberaceae: *Alpinia* Roxb., *Boesenbergia* Kuntze, *Globba* L., *Hedychium* J.Koenig, *Larsenianthus* W.J.Kress & Mood and *Zingiber* Mill. (Table 1). Interestingly, pseudovivipary (bulbil formation) was one of the key characters used to distinguish section Globba into two series by Schumann (1904) during the revision of Zingiberaceae, although recent molecular studies have not addressed the role of bulbil as an important character defining clades (see Williams et al. 2004).

**Table 1.** Summary of published and personal records where vivipary *sensu lato* were identified within Zingiberaceae. All *Globba* synonyms following Williams et al. (2004), Newman and Pullan (2007), WCSP (2018).

Vivipary recorded in Zingiberaceae	Type of vivipary	Reference(s)
*Alpinia* Roxb.		
*A. mutica* Roxb.	Vivipary*	Aswani and Sabu 2015
*A. purpurata* (Vieill.) K.Schum.	Pseudovivipary	Dekkers et al. 1991
*Boesenbergia* Kuntze		
*B. parvula* (Wall. ex Baker) Kuntze	Pseudovivipary	Mood et al. 2016
*B. pulcherrima* (Wall.) Kuntze	Pseudovivipary	Aishwarya and Sabu 2015
*Camptandra* Ridl.		
*C. latifolia* Ridl.	Vivipary*	Ridley 1899
*Curcuma* L.		
*C. coriacea* Mangaly & M.Sabu	Vivipary*	Leong-Škorničková 2007
*Globba* L.		
*G. aurantiaca* Miq.	Pseudovivipary	Ridley 1899
*G. bicolor* Gagnep.	Pseudovivipary	Gagnepain 1901
*G. bulbifera* Roxb.	Pseudovivipary	Horaninow 1862, Baker 1894
*G. cambodgensi*s Gagnep.	Pseudovivipary	Gagnepain 1901
*G. cernua* Baker Synonym: *G. brachycarpa* Baker or *G. trachycarpa* Baker	Pseudovivipary	Schumann 1904, Box and Rudall 2006
*G. chinensis* K.Schum.	Pseudovivipary	Schumann 1904
*G. colpicola* K.Schum.	Pseudovivipary	Schumann 1904
*G. lancangensis* Y.Y.Qian	Pseudovivipary	Zhou et al. 2007
*G. leucantha* Miq. Synonym: *G. pallidiflora* Baker ex Ridl.	Pseudovivipary	Ridley 1899
*G. marantina* L. Synonyms: *G. barthei* Gagnep., *G. ectobolos* K.Schum., *G. heterobractea* K.Schum., *G. strobilifera* Zoll. & Moritzi	Pseudovivipary	Horaninow 1862, Gagnepain 1901, Liu et al. 2004, Williams et al. 2004, Box and Rudall 2006
*G. multiflora* Wall. Synonym: *G. rubromaculata* J.Lal & D.M.Verma	Pseudovivipary	Baker 1894, Schumann 1904, This paper
*G. parva* Gagnep.	Pseudovivipary	Gagnepain 1901, Schumann 1904
*G. pendula* Roxb. Synonyms: *G. calophylla* Ridl., *G. kingii* Baker, *G. panicoides* Miq., *G. stenothyrsa* Baker, *G. wallichii* Baker	Pseudovivipary	Ridley 1899
*G. platystachya* Baker	Pseudovivipary	Baker 1894
*G. racemosa* Sm. Synonyms: *G. clarkei* Baker, *G. hookeri* C.B.Clarke ex Baker	Pseudovivipary	Baker 1894, Schumann 1904
*G. ranongensis* Picheans. & Tiyawora.	Pseudovivipary	Picheansoonthon and Tiyaworanant 2010
*G. schomburgkii* Hook.f. Synonym: *G. globulifera* Gagnep.	Pseudovivipary	Gagnepain 1901, Schumann 1904
*G. sessiliflora* Sims Synonyms: *G. canarensis* Baker, *G. careyana* Roxb., *G. ophioglossa* Wight	Pseudovivipary	Horaninow 1862, Baker 1894, Schumann 1904
*G. substrigosa* Synonym: *G. aphanantha* K.Larsen	Pseudovivipary	Larsen et al. 1998
*G. unifolia* Ridl.	Pseudovivipary	Holttum 1950
*G. ustulata* Gagnep.	Pseudovivipary	Gagnepain 1901
*Hedychium* J.Koenig		
*H. greenii* W.W.Sm.	Pseudovivipary	Smith 1911
*H. marginatum* C.B.Clarke	Facultative vivipary	This paper
H. speciosum var. gardnerianum (Ker Gawl.) Sanoj & M.Sabu Synonym: *H. gardnerianum* Sheppard ex Ker Gawl.	Facultative vivipary	Djeddour et al. 2012, Bhadra et al. 2013, This paper
*H. thyrsiforme* Buch.-Ham. ex Sm.	Facultative vivipary	This paper
*H. urophyllum* G.Lodd.	Facultative vivipary	This paper
*Hornstedtia* Retz.		
*H. scyphifera* J.Koenig ex Steud.	Vivipary*	Leong-Škorničková (pers. comm.)
*Larsenianthus* W.J.Kress & Mood		
*L. careyanus* (Benth. & Hook.f.) W.J.Kress & Mood	Pseudovivipary	Mibang and Das 2017, Poulsen (pers. comm.), This paper
*Zingiber* Mill.		
*Z. puberulum* Ridl.	Pseudovivipary	Leong-Škorničková (pers. comm.)
*Z. singapurense* Škorničk.	Pseudovivipary	Leong-Škorničková et al. 2014

*Possibly, Facultative vivipary

**Figure 1.**
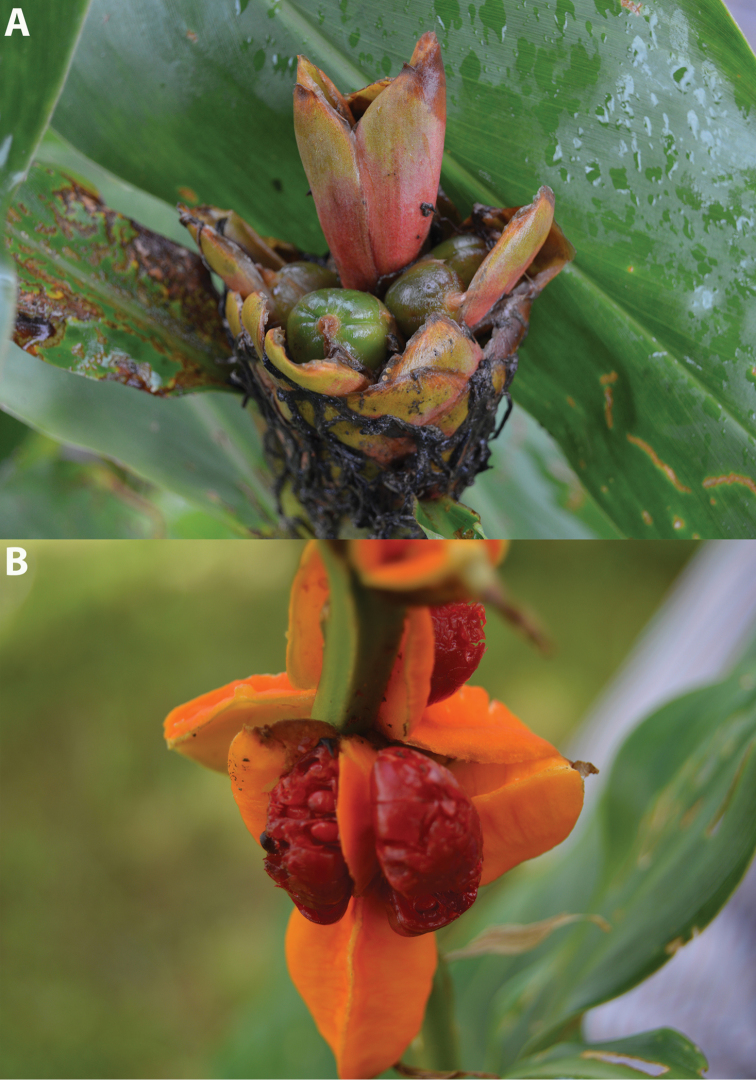
Septifragal capsule of *Hedychium*. **A** Unripe fruit of *H. marginatum* **B** Ripe fruit of *H. spicatum* (seeds can be seen covered by aril). Photographed by A. Ashokan.

The genus *Hedychium* (more than 80 species, see Sanoj 2011) is native to the Indian subcontinent, China, Southeast Asia (mainland and maritime) and Madagascar (Kress et al. 2002, Newman et al. 2004, Newman and Pullan 2007, Sanoj et al. 2013, WCSP 2018). The highly speciose regions are identified to be NE India (Arunachal Pradesh, Manipur, Meghalaya, Mizoram, Nagaland and Sikkim), Southwest China (Yunnan), Myanmar, Thailand, Borneo and Java (Holttum 1950, Sirirugsa and Larsen 1995, Gao et al. 2008, Sarangthem et al. 2013). The plant habit ranges from terrestrial to epiphytic as well as lithophytic and each plant has a perennial rhizome which bears new ramets annually. Each ramet gives rise to a terminal inflorescence (known as thyrse) which consists of primary inflorescence bracts that hold flowers in cincinni (Kirchoff 1997). Two main types of inflorescence bracts are identified in *Hedychium*- imbricate (where bracts are broad and overlapping, hiding the rachis) and folded (where bracts are narrow and fold partially or completely to enclose the flowers, leaving rachis visible, Holttum 1950). The flowers are hermaphroditic, characterised by reduced petals and petaloid staminodes. The fruit is a septifragal capsule, light to dark green when young and changes to yellow with age (Fig. 1A). It splits open into three fleshy lobes, deep yellow or orange internally, with seeds arranged as in axile placentation. Mature seed is brown or black, covered by an aril which is either deep red or bluish-violet (in *H. hookeri* Baker, Srivastava 1984; fig. 1B). Frugivorous birds (Orchard 1973, Larsen et al. 1998) and rodents (Ridley 1899, Shiels 2011) are major dispersers of *Hedychium* seeds.

Although the name *Hedychium* is more than 230 years old (Koenig 1783), the report of vivipary in this genus is very recent (Djeddour et al. 2012, Bhadra et al. 2013). The first vivipary in *Hedychium* was detailed by Bhadra et al. (2013) in *H. elatum* from Kalimpong, on the way to Darjeeling (West Bengal, India) although it had already been reported in Djeddour et al. (2012). Here, four new observations are reported of facultative vivipary in the genus *Hedychium*: *H. marginatum*, H. speciosum var. gardnerianum, *H. thyrsiforme* and *H. urophyllum*. The taxonomic misidentification in Bhadra et al. 2013 is also reported and it is suggested that vivipary was not observed by them in *H. elatum* but the plant observed by them could likely be H. speciosum var. gardnerianum. Since voucher information is not available from Bhadra et al. (2013), their published images were used to assign an identity to the plant identified by them as *H. elatum*. Based on a published image (figure 1A in Bhadra et al. 2013) and the characters listed here in Table 2, it is clear that the species they reported cannot be *H. elatum*. Since voucher specimens or high resolution images are critical in species-level identification in *Hedychium*, it is suggested that the two likely species illustrated by Bhadra et al. in their figure 1A are either *H. gracile* Roxb. or *H. griffithianum* Wall. (Wallich 1853) based on the inflorescence shape and delicate nature of the inflorescence bracts and flowers, which are characteristics to these species (Table 2). Further, the plant shown in figures 1B–H of Bhadra et al. is identified as H. speciosum var. gardnerianum (Ker Gawl.) Sanoj & M.Sabu. Therefore, the plant image shown with vivipary (figures 1B–H in Bhadra et al. 2013) is identified as H. speciosum var. gardnerianum because of the taxonomic description given by Bhadra et al. as “*leaves white pulverulent beneath*” which is characteristic of H. speciosum var. gardnerianum (Baker 1894).

**Table 2.** List of characters used to resolve the taxonomic identifications of plants in Figures 1A–H in Bhadra et al. 2013. Characters in bold were used by them to describe *H. elatum*, which are characteristics of either *H. gracile* or *H. griffithianum* or H. speciosum var. gardnerianum.

Taxon	Nature of lamina (abaxial)	Midrib type (adaxial)	Inflorescence height (cm)	Inflorescence density	Colour (labellum and filament)	Length of corolla segments (cm)
*H. elatum*	not pulverulent	faintly grooved	>30	dense to moderately dense	labellum pinkish-white with red centre; filament reddish-pink	3.5
*H. gracile*	not pulverulent	faintly grooved	5–10	**lax to moderately dense**	labellum creamy white with pale red base; filament bright red	**2.5**
*H. griffithianum*	not pulverulent	faintly grooved	15–22	moderately dense	labellum creamy white with pale red base; filament bright red	**2.5**
H. speciosum var. gardnerianum	**pulverulent**	**deeply grooved**	30–45	dense to moderately dense	labellum lemon yellow; filament bright red	2.5–3.6

For taxonomic clarity, the key morphological and ecological characters of the *Hedychium* taxa) are listed below where facultative vivipary has been observed. Voucher information is provided below as: collector name, voucher number, herbarium deposited.

**Figure 2.**
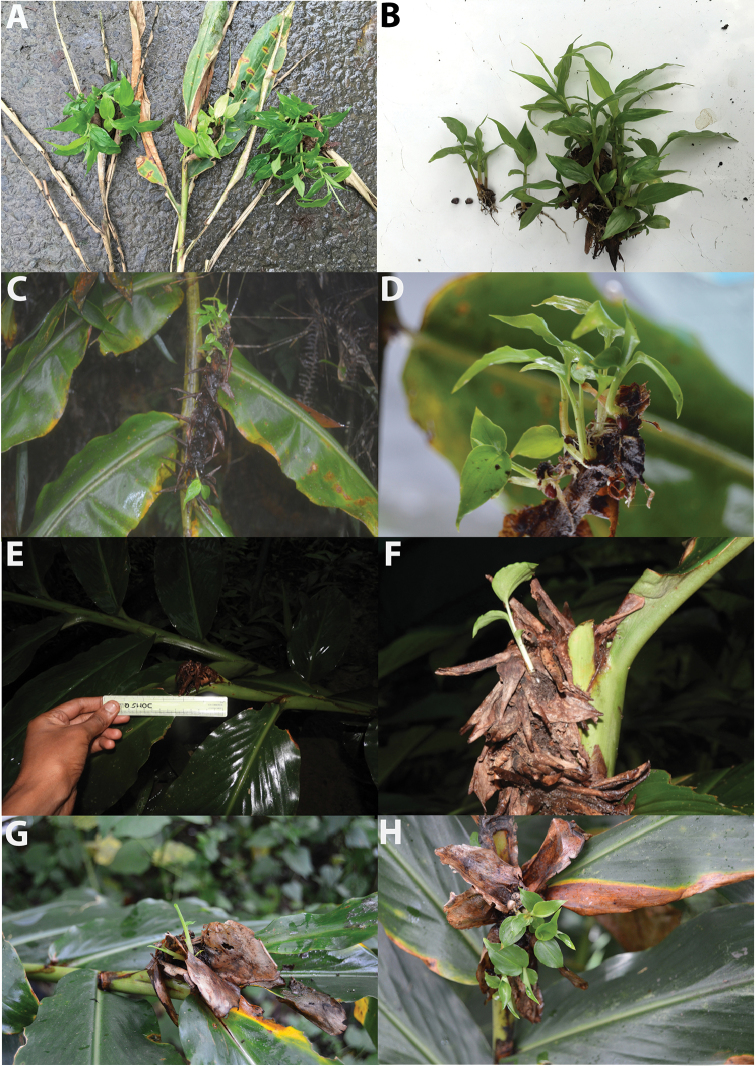
Facultative vivipary in *Hedychium*. **A–B** *Hedychium marginatum* **C–D** H. speciosum var. gardnerianum, **E**&**F.** *H. thyrsiforme*, **G**&**H.** *H. urophyllum*. Photographed by A. Ashokan & N.S. Prasanna (E&F).

**Figure 3.**
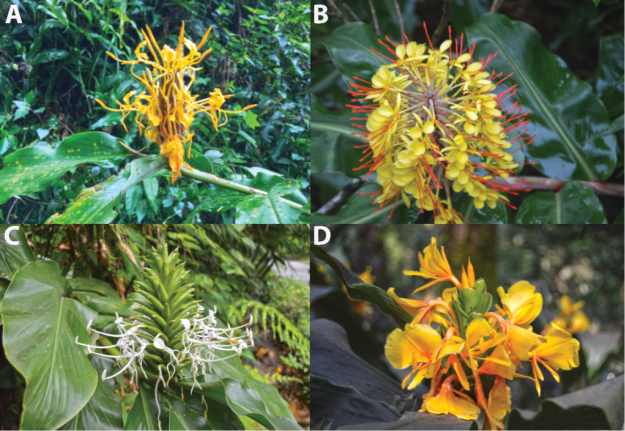
Inflorescence. **A** *Hedychium marginatum* **B** H. speciosum var. gardnerianum **C** *H. thyrsiforme* **D** *H. urophyllum*. Photographed by A. Ashokan.

1. *Hedychium marginatum* C.B.Clarke (Figs 2A–B, 3A; V. Gowda, VG-NL1899, BHPL)

**Figure 4.**
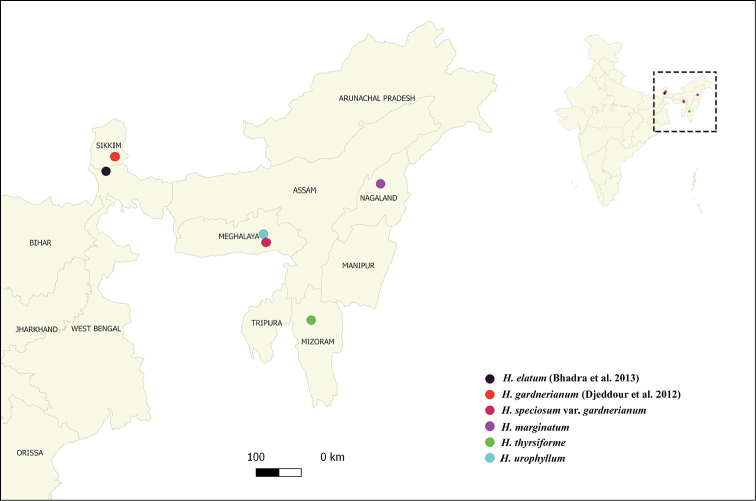
Distribution records for facultative vivipary in *Hedychium* in NE India.

Plant terrestrial, up to 1.5 m tall. Inflorescence conical; bracts imbricate; cincinni 3–6 flowers. Flowers orange-yellow, fragrant. Capsule elliptic, green and minutely hairy. Seeds many; aril red. The viviparous individual (bearing at least 10 seedlings on its dried infructescence) was observed along the Mokokchung-Tuensang Road, Mokokchung, Nagaland (26°19'59"N; 94°32'50"E) in August 2017 (Fig. 4).

It is very common in the Indian states of Manipur and Nagaland (Clarke 1890, Baker 1894, Sanoj 2011).

Flowering period: August to October; fruiting from September to November.

2. Hedychium speciosum var. gardnerianum (Ker Gawl.) Sanoj & M.Sabu (Figs 2C, D, 3B; A. Ashokan, VG-ML1660, BHPL)

Plant terrestrial, up to 2.5 m tall. Inflorescence cylindrical; bracts folded, folding supervolute; cincinni 2 flowers. Flowers lemon-yellow with bright red stamen, highly fragrant. Capsule oblong, green and glabrous. Seeds many; aril red. The viviparous individuals were found growing along the Shillong-Dawki Road in the East Khasi Hills district, Meghalaya (25°22'25"N; 91°52'37"E) in August 2016 and 2017 (Fig. 4).

It has a wide distribution range covering Eastern Himalaya and NE India, especially the Indian states of Arunachal Pradesh and Meghalaya (Edwards 1823, Roscoe 1828).

Flowering period: July to October; fruiting from September to November.

3. *Hedychium thyrsiforme* Buch.-Ham. ex Sm. (Figs 2E–F, 3C; N.S.Prasanna, VG-MZ2201, BHPL)

Plant terrestrial, up to 1.5 m tall. Inflorescence pyramidal; bracts folded, folding supervolute; cincinni 3–8 flowers. Flowers white, mildly fragrant. Capsule oblong, green and glabrous. Seeds many; aril red. The viviparous individual (bearing one seedling on its dried infructescence) was observed in Aizawl, Mizoram (23°43'48"N; 92°43'52"E) in July 2017 (Fig. 4).

It grows very commonly in the Indian state of Mizoram. It was originally described from Nepal (Smith 1811).

Flowering period: August to October; fruiting from September to November.

4. *Hedychium urophyllum* G.Lodd. (Figs 2G–H, 3D; A. Ashokan, VG-ML1612 & 1725, BHPL)

Plant terrestrial, up to 2.2 m tall. Inflorescence conical; bracts imbricate; bracts are found to retain water; cincinni 2–4 flowers. Flowers bright yellow, highly fragrant. Capsule elliptic, dark green and glabrous. Seeds many; aril red. The viviparous *H. urophyllum* was growing in a mixed pine forest at Sanmer along the Shillong-Elephant Falls Road, East Khasi Hills district, Meghalaya (25°32'47"N; 91°50'16"E) in June 2015 and August 2016 (Fig. 4).

It is endemic to Meghalaya (Loddiges 1831, Sanoj 2011).

Flowering period: July to September; fruiting from September to November.

## Discussion

During the three year study in NE India, four different *Hedychium* taxa showing vivipary have been identified: *H. marginatum*, H. speciosum var. gardnerianum, *H. thyrsiforme* and *H. urophyllum*. However, current observations of vivipary in *Hedychium* cannot be strictly identified as true vivipary or cryptovivipary (Elmqvist and Cox 1996, Cota-Sánchez 2004) since the fruit of *Hedychium* (septifragal capsule) dehisces before seed maturation while true vivipary requires the embryo to penetrate the fruit pericarp during germination. This makes the viviparous seed germination in *Hedychium* or gingers a case of “facultative vivipary”, which is introduced for the first time in gingers. The term “facultative vivipary” is proposed for the reproductive strategy observed in *Hedychium* since it represents the facultative nature of this reproductive strategy (occurs only when seeds are retained by bracts) as well as the viviparous nature of the germinating seed (seedling borne on the parent plant).

The factors leading to the incidence of facultative vivipary can be identified as a combination of climatic, ecological and physiological variables (Lee and Harmer 1980, Cox and Humphries 1993). In most *Hedychium*, flowering and fruiting coincide with the rainy season (an ecological trait shared amongst almost all *Hedychium*). The morphology of bracts in many *Hedychium* is such that the bracts can hold water. Thus the bracts provide a platform for the germination of the recalcitrant seeds by holding water which favours their germination. Further, these bracts are retained even after maturity of seeds thus facilitating their continued growth as seedlings. Thus, facultative vivipary may be a result of ‘failure to disperse’ either due to the structural hindrance imparted by the bracts or due to the absence of a disperser. The importance of facultative vivipary was not discounted as an important reproductive strategy that may facilitate seed germination under unpredictable climatic conditions, but this remains to be tested.

This multi-year observation of facultative vivipary in four *Hedychium* taxa suggests that facultative vivipary may be a more common reproductive strategy than that which is known so far. The immediate germination of seeds on the parent plant in at least four taxa also implies that recalcitrance of seeds (i.e. absence of dormancy) may be common in *Hedychium* (also true for other gingers, Leong-Škorničková, pers. com.). Recalcitrance has been suggested to be a common characteristic of plants growing in wet habitats (Farnsworth 2000), which is true for most *Hedychium*. Further, morphologically, it has been shown that *Hedychium* and other gingers have a thin seed coat (Benedict et al. 2015), which may also facilitate recalcitrance. Thus, it is predicted that facultative vivipary may be more common in *Hedychium* because of the physiology of the seed, i.e. its recalcitrant nature.

**Figure 5.**
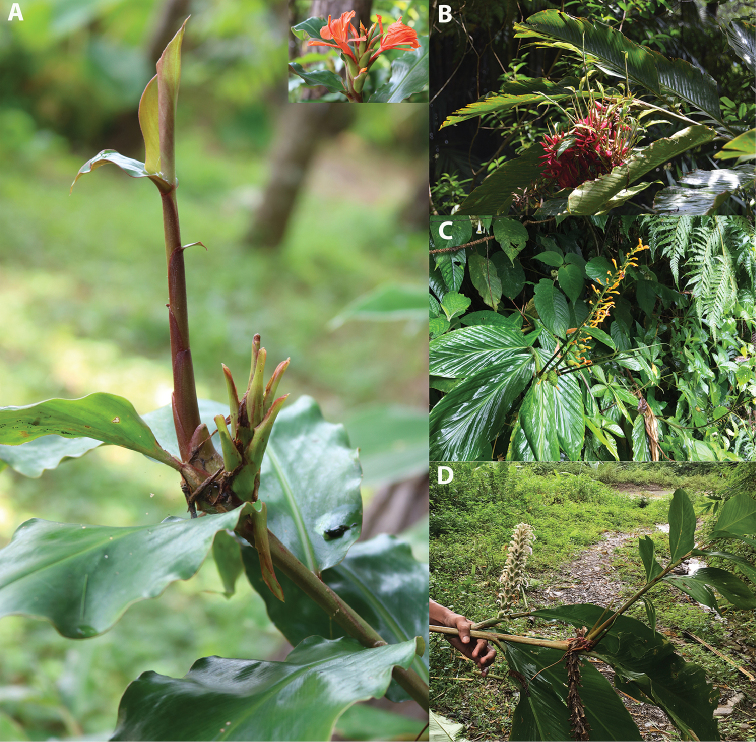
Pseudovivipary (Bulbil formation). **A** *Hedychium greenii* (the inset in the upper right is an inflorescence) **B** *Alpinia purpurata* **C** *Globba multiflora* **D** *Larsenianthus careyanus*. Photographed by A. Ashokan & Preeti (A).

The authors’ efforts to summarise vivipary *sensu lato* across Zingiberaceae from published literature showed that vivipary *sensu stricto* is absent and that all reported cases of vivipary in other gingers can also only be identified as “facultative vivipary” or pseudovivipary, as in the case of *Hedychium*. Other genera that display facultative vivipary (except in *A. mutica*) also show similar characters as observed in *Hedychium* that favour this reproductive strategy, such as persistent bracts, bract structure that may retain the seeds, and recalcitrant seeds. It was found that both facultative vivipary and pseudovivipary are common amongst gingers with pseudovivipary being more frequent only in *Globba* (Table 1). Finally, in the genus *Hedychium*, pseudovivipary is only reported in *H. greenii* (Fig. 5).

To understand if vivipary *sensu lato* is a convergent character in *Hedychium*, different types of vivipary were mapped on a preliminary molecular phylogenetic tree (modified from nrDNA ITS tree of Wood et al. 2000, with the addition of *H. marginatum* and H. speciosum var. gardnerianum). It was found that both facultative vivipary and pseudovivipary are currently restricted only to a few members within the Clade IV (as defined in Wood et al. 2000). To understand the relevance of facultative vivipary and pseudovivipary as important reproductive strategies in gingers, it is suggested that future studies should treat different types of vivipary as an important morphological character along with seed characteristics (i.e. presence or absence of dormancy).

## References

[B1] Aishwarya K Sabu M (2015) *Boesenbergia pulcherrima* and *B. tiliifolia* (Zingiberaceae) in India: notes on the identity, variability and typification. Rheedea 25(1): 59–68.

[B2] Aswani K Sabu M (2015) Reproductive biology of *Alpinia mutica* Roxb. (Zingiberaceae) with special reference to flexistyly pollination mechanism. The International Journal of Plant Reproductive Biology 7(1): 48–58.

[B3] Baker JG (1894) Scitamineae. In: Hooker JD (Ed.) Flora of British India. L. Reeve & Co., London, 6, 198–264.

[B4] Benedict JC Smith SY Collinson ME Leong-Škorničková J Specht CD Marone F Xiao X Parkinson DY (2015) Seed morphology and anatomy and its utility in recognizing subfamilies and tribes of Zingiberaceae. American Journal of Botany 102(11): 1814–1841. https://doi.org/10.3732/ajb.1500300 26507111 10.3732/ajb.1500300

[B5] Bhadra S Ghosh M Mukherjee A Bandyopadhyay M (2013) Vivipary in *Hedychium elatum* (Zingiberaceae). Phytotaxa 130(1): 55–59. https://doi.org/10.11646/phytotaxa.130.1.7

[B6] Box MS Rudall PJ (2006) Floral structure and ontogeny in *Globba* (Zingiberaceae). Plant Systematics and Evolution 258(1): 107–122. https://doi.org/10.1007/s00606-005-0395-4

[B7] Clarke CB (1890) Plants of Kohima and Moneypore. Journal of Linnean Society (Botany) 25: 75.

[B8] Cota-Sánchez JH (2004) Vivipary in the Cactaceae: its taxonomic occurrence and biological significance. Flora-Morphology, Distribution, Functional Ecology of Plants 199(6): 481–490. https://doi.org/10.1078/0367-2530-00175

[B9] Cota-Sánchez JH Abreu DD (2007) Vivipary and offspring survival in the epiphytic cactus *Epiphyllum phyllanthus* (Cactaceae). Journal of Experimental Botany 58(14): 3865–3873. https://doi.org/10.1093/jxb/erm232 17975210 10.1093/jxb/erm232

[B10] Cox PA Humphries CJ (1993) Hydrophilous pollination and breeding system evolution in seagrasses: a phylogenetic approach to the evolutionary ecology of the Cymodoceaceae. Botanical Journal of the Linnean Society 113: 217–226. https://doi.org/10.1111/j.1095-8339.1993.tb00338.x

[B11] Dekkers AJ Rao AN Goh CJ (1991) In vitro storage of multiple shoot cultures of gingers at ambient temperatures of 24–29°C. Scientia Horticulturae 47(1/2):157–167. https://doi.org/10.1016/0304-4238(91)90037-Y

[B12] Djeddour DH Pratt C Shaw RH (2012) The potential for the biological control of *Hedychium gardnerianum* A report of the 4th Phase Research on the Biological Control of *Hedychium gardnerianum*. Produced for Landcare Research, New Zealand and The Nature Conservancy, Hawai’i, 16–17.

[B13] Edwards S (1823) The Botanical Register 9: 774.

[B14] Elmqvist T Cox PA (1996) The evolution of vivipary in flowering plants. Oikos 77(1): 3–9. https://doi.org/10.2307/3545579

[B15] Farnsworth E (2000) The ecology and physiology of viviparous and recalcitrant seeds. Annual Review of Ecology and Systematics 31(1): 107–138. https://doi.org/10.1146/annurev.ecolsys.31.1.107

[B16] Gagnepain MF (1901) Révision des genres *Mantisia* et *Globba* (Zingibérées) de l’herbier du muséum. Bulletin de la Société Botanique de France 48(3): 201–216. https://doi.org/10.1080/00378941.1901.10831839

[B17] Gao L Liu N Huang B Hu X (2008) Phylogenetic analysis and genetic mapping of Chinese *Hedychium* using SRAP markers. Scientia Horticulturae 117(4): 369–377. https://doi.org/10.1016/j.scienta.2008.05.016

[B18] Goebel KE (1905) Organography of Plants. Hafner, New York.

[B19] Holttum RE (1950) The Zingiberaceae of the Malay Peninsula. Gardens’ Bulletin Singapore 13: 1–249.

[B20] Horaninow PF (1862) Prodromus Monographiae Scitaminarum. St. Petersburg.

[B21] Kirchoff BK (1997) Inflorescence and flower development in the *Hedychieae* (Zingiberaceae): *Hedychium*. Canadian Journal of Botany 75(4): 581–594. https://doi.org/10.1139/b97-065

[B22] Koenig JG (1783) Descriptions Monandrarum pro annis. In: Retzius AJ (Ed.) Observationes Botanicae. Lipsiae, 3, 45–76.

[B23] Kress WJ Prince LM Williams KJ (2002) The phylogeny and a new classification of the gingers (Zingiberaceae): evidence from molecular data. American Journal of Botany 9(10): 1682–1696. https://doi.org/10.3732/ajb.89.10.1682 10.3732/ajb.89.10.1682 21665595

[B24] Larsen K Lock JM Maas H Maas PJ (1998) Zingiberaceae. In: Kubitzki K (Ed.) The Families and Genera of Vascular Plants IV. Flowering Plants: Monocotyledons. Alismatanae and Commelinanae (except Gramineae). Springer, Berlin, Heidelberg, 474–495. https://doi.org/10.1007/978-3-662-03531-3_49

[B25] Law R Cook RE Manlove RJ (1983) The ecology of flower and bulbil production in *Polygonum viviparum*. Nordic Journal of Botany 3(5): 559–566. https://doi.org/10.1111/j.1756-1051.1983.tb01468.x

[B26] Lee JA Harmer R (1980) Vivipary, a reproductive strategy in response to environmental stress? Oikos 35(2): 254–265. https://doi.org/10.2307/3544433

[B27] Leong-Škorničková J (2007) Taxonomic Studies in Indian *Curcuma* L. PhD Thesis, Charles University, Prague.

[B28] Leong-Škorničková J Thame A Chew PT (2014) Notes on Singapore native Zingiberales I: A new species of *Zingiber* and notes on the identities of two further *Zingiber* taxa. Gardens’ Bulletin Singapore 66: 153–167.

[B29] Liu Z Chen J Bai Z (2004) Comparative studies on reproductive mechanisms of three species in *Globba* (Zingiberaceae). Wuhan Botanical Research 22(2): 145–152.

[B30] Loddiges CL (1831) No. 1785. *Hedychium urophyllum*. The Botanical Cabinet: consisting of coloured delineations of plants, from all countries, with a short account of each, directions for management.

[B31] Mibang T Das AK (2017) Taxonomic Investigation on Genus *Larsenianthus* (Zingiberaceae) of Siang Valley, Arunachal Pradesh. Bulletin of Arunachal Forest Research 32(1–2): 41–48.

[B32] Mood JD Tanaka N Aung MM Murata J (2016) The genus *Boesenbergia* (Zingiberaceae) in Myanmar with two new records. Gardens’ Bulletin Singapore 68(2): 299–318. https://doi.org/10.3850/S2382581216000235

[B33] Newman M Lhuillier-Chaigneau A Poulsen AD (2004) Checklist of the Zingiberaceae of Malesia. Blumea Suppl. 16: 1–166.

[B34] Newman M Pullan M (2007) Zingiberaceae Resource Centre. Edinburgh: Royal Botanic Garden. http://rbg-web2.rbge.org.uk/ZRC/home.html [accessed 18 February 2018[

[B35] Ofir M Kigel J (2014) Temporal and intraclonal variation of flowering and pseudovivipary in *Poa bulbosa*. Annals of Botany 113(7): 1249–1256. https://doi.org/10.1093/aob/mcu037 24685715 10.1093/aob/mcu037 PMC4030809

[B36] Orchard AE (1973) Zingiberaceae in New Zealand. Records of the Auckland Institute and Museum 10: 109–117.

[B37] Picheansoonthon C Tiyaworanant S (2010) A New Species of *Globba* (Zingiberaceae) from Southern Thailand. Journal of Japanese Botany 85(1): 25–29.

[B38] Poulsen AD Nordal I (2005) A phenetic analysis and revision of Guineo-Congolean rainforest taxa of *Chlorophytum* (Anthericaceae). Botanical Journal of the Linnean Society 148(1): 1–20. https://doi.org/10.1111/j.1095-8339.2005.00386.x

[B39] Ridley HN (1899) The Scitamineae of the Malay Peninsula. Journal of the Straits Branch of the Royal Asiatic Society 32: 85–184.

[B40] Roscoe W (1828) Monandrian Plants of the Order Scitamineae. Liverpool.

[B41] Sanoj E (2011) Taxonomic Revision of the Genus *Hedychium* J. Koenig (Zingiberaceae) in India. PhD Thesis, University of Calicut, Kerala.

[B42] Sanoj E Sabu M Pradeep AK (2013) Circumscription and lectotypification of *Hedychium villosum* and its variety H. villosum var. tenuiflorum (Zingiberaceae). PhytoKeys 25: 75–85. https://doi.org/10.3897/phytokeys.25.4113 10.3897/phytokeys.25.4113 PMC3819131 24198714

[B43] Sarangthem N Talukdar NC Thongam B (2013) Collection and evaluation of *Hedychium* species of Manipur, Northeast India. Genetic resources and crop evolution 60(1): 13–21. https://doi.org/10.1007/s10722-012-9810-1

[B44] Schumann K (1904) Zingiberaceae, IV, 46. In: Engler A (Ed.) Das Pflanzenreich, Heft 20. Leipzig, Berlin, 1–458.

[B45] Shiels AB (2011) Frugivory by introduced black rats (*Rattus rattus*) promotes dispersal of invasive plant seeds. Biological Invasions 13: 781–792. https://doi.org/10.1007/s10530-010-9868-7

[B46] Sirirugsa P Larsen K (1995) The genus *Hedychium* (Zingiberaceae) in Thailand. Nordic Journal of Botany 15(3): 301–304. https://doi.org/10.1111/j.1756-1051.1995.tb00156.x

[B47] Smith JE (1811) *Hedychium*. In: Rees A (Ed.) Cyclopaedia. Vol .17. London.

[B48] Smith WW (1911) Some additions to the Flora of Eastern Himalaya. Records of the Botanical Survey of India 4: 261–272.

[B49] Srivastava SC (1984) A Taxonomic Study of Genus *Hedychium* Koen. (Zingiberaceae) in India and its Vicinity. PhD Thesis, University of Calcutta, West Bengal.

[B50] Wallich N (1853) Initiatory attempt to define the species of *Hedychium*, and settle their synonymy. Hooker’s Journal of Botany and Kew Garden Miscellany 5: 367–377.

[B51] WCSP (2018) World Checklist of Selected Plant Families. Facilitated by the Royal Botanic Gardens, Kew. Published on the Internet. http://wcsp.science.kew.org/ [Retrieved 18 February 2018]

[B52] Williams KJ Kress WJ Manos PS (2004) The phylogeny, evolution, and classification of the genus *Globba* and tribe Globbeae (Zingiberaceae): Appendages do matter. American Journal of Botany 91: 100–114. https://doi.org/10.3732/ajb.91.1.100 21653367 10.3732/ajb.91.1.100

[B53] Wood TH Whitten WM Williams NH (2000) Phylogeny of *Hedychium* and related genera (Zingiberaceae) based on ITS sequence data. Edinburgh Journal of Botany 57(2): 261–270. https://doi.org/10.1017/S0960428600000196

[B54] Zhou H Chen J Chen F (2007) Ant-mediated seed dispersal contributes to the local spatial pattern and genetic structure of *Globba lancangensis* (Zingiberaceae). Journal of Heredity 98(4): 317–324. https://doi.org/10.1093/jhered/esm032 17602181 10.1093/jhered/esm032

